# Comparative Assessment of Filtration- and Precipitation-Based Methods for the Concentration of SARS-CoV-2 and Other Viruses from Wastewater

**DOI:** 10.1128/spectrum.01102-22

**Published:** 2022-08-11

**Authors:** Kata Farkas, Cameron Pellett, Natasha Alex-Sanders, Matthew T. P. Bridgman, Alexander Corbishley, Jasmine M. S. Grimsley, Barbara Kasprzyk-Hordern, Jessica L. Kevill, Igor Pântea, India S. Richardson-O’Neill, Kathryn Lambert-Slosarska, Nick Woodhall, Davey L. Jones

**Affiliations:** a Centre for Environmental Biotechnology, School of Natural Sciences, Bangor University, Bangor, Gwynedd, United Kingdom; b School of Ocean Sciences, Bangor University, Anglesey, United Kingdom; c Royal (Dick) School of Veterinary Studies and The Roslin Institute, University of Edinburgh, Roslin, United Kingdom; d UK Health Security Agency, Environmental Monitoring for Health Protection, London, United Kingdom; e Department of Chemistry, University of Bath, Bath, United Kingdom; f Food Futures Institute, Murdoch University, Murdoch, Western Australia, Australia; McGill University

**Keywords:** enteric viruses, environmental virology, human respiratory viruses, public health surveillance, sewage concentration

## Abstract

Wastewater-based epidemiology (WBE) has been widely used to track levels of SARS-CoV-2 infection in the community during the COVID-19 pandemic. Due to the rapid expansion of WBE, many methods have been used and developed for virus concentration and detection in wastewater. However, very little information is available on the relative performance of these approaches. In this study, we compared the performance of five commonly used wastewater concentration methods for the detection and quantification of pathogenic viruses (SARS-CoV-2, norovirus, rotavirus, influenza, and measles viruses), fecal indicator viruses (crAssphage, adenovirus, pepper mild mottle virus), and process control viruses (murine norovirus and bacteriophage Phi6) in laboratory spiking experiments. The methods evaluated included those based on either ultrafiltration (Amicon centrifugation units and InnovaPrep device) or precipitation (using polyethylene glycol [PEG], beef extract-enhanced PEG, and ammonium sulfate). The two best methods were further tested on 115 unspiked wastewater samples. We found that the volume and composition of the wastewater and the characteristics of the target viruses greatly affected virus recovery, regardless of the method used for concentration. All tested methods are suitable for routine virus concentration; however, the Amicon ultrafiltration method and the beef extract-enhanced PEG precipitation methods yielded the best recoveries. We recommend the use of ultrafiltration-based concentration for low sample volumes with high virus titers and ammonium levels and the use of precipitation-based concentration for rare pathogen detection in high-volume samples.

**IMPORTANCE** As wastewater-based epidemiology is utilized for the surveillance of COVID-19 at the community level in many countries, it is crucial to develop and validate reliable methods for virus detection in sewage. The most important step in viral detection is the efficient concentration of the virus particles and/or their genome for subsequent analysis. In this study, we compared five different methods for the detection and quantification of different viruses in wastewater. We found that dead-end ultrafiltration and beef extract-enhanced polyethylene glycol precipitation were the most reliable approaches. We also discovered that sample volume and physico-chemical properties have a great effect on virus recovery. Hence, wastewater process methods and start volumes should be carefully selected in ongoing and future wastewater-based national surveillance programs for COVID-19 and beyond.

The COVID-19 pandemic has so far resulted in 410 million confirmed cases and 5.8 million deaths worldwide (1). The illness is caused by SARS-CoV-2, an enveloped, spherical coronavirus with a single-stranded RNA (ssRNA) genome that is 30 kb in size (2). COVID-19 causes a wide range of symptoms, including flu-like symptoms, such as fever and chills, cough, fatigue, headache, and the loss of taste and smell (3). The symptoms are often mild, and the infected individuals may be asymptomatic; however, they can still infect others (4). These cases are usually undetected, which largely contributes to the rapid spread of the disease (5).

Wastewater-based epidemiology (WBE) has been successfully used for the surveillance of chemicals and pathogens, including several human viruses at the community level (6–8). Since the beginning of the COVID-19 pandemic, many countries have trialed the usefulness of WBE for tracking SARS-CoV-2 at the community level. As all infected individuals, including asymptomatic and presymptomatic cases, shed the virus in their feces, the monitoring of wastewater can be a useful addition to COVID-19 surveillance (9). Several studies have shown that the RNA of the virus can be readily detected and quantified in wastewater and that WBE can be used as an early warning system and a predictive tool for COVID-19 monitoring (9–12). Hence, many countries have implemented WBE as a component of their COVID-19 surveillance and decision-making portfolio (13–18).

Although WBE is a cost-effective approach supporting the understanding of viral disease spread, it has its limitations. A major factor affecting the use of WBE is the robust detection of the target virus in the samples. As the viral RNA is usually present at low concentrations in wastewater, the samples need to be concentrated prior to nucleic acid extraction and the quantification of the viral target using quantitative or digital PCR (19). Many methods have been used for wastewater concentration for SARS-CoV-2 RNA quantification, including ultracentrifugation, filtration, ultrafiltration, adsorption and precipitation-based approaches (20–22). However, the viral recovery using these methods is usually not assessed (23–25).

There are many factors to consider when selecting a concentration method for WBE, including the availability of equipment, cost, time available for sample processing, optimal sample volume, etc. As ultracentrifugation is time-consuming and requires expensive equipment, it has not been implemented in routine WBE surveillance. Electronegative and electropositive membrane filtration methods seem a viable alternative; however, extracting viral nucleic acids from a membrane filter can be challenging (26). Furthermore, the filter membranes are subject to clogging if the samples have a high turbidity. Nonetheless, precipitation- and ultrafiltration-based approaches are more robust and versatile and hence may be more suitable for many WBE applications (27–29).

In this study, we evaluated the usefulness of wastewater concentration methods for the surveillance of pathogenic viruses, namely, SARS-CoV-2, influenza, measles virus (MeV), norovirus (NoV), rotavirus (RoV), fecal indicator viruses, such as crAssphage, human mastadenoviruses (AdV), and pepper mild mottle virus (PMMoV), and potential process control viruses, including Phi6 bacteriophage and murine norovirus. We explored the viral recoveries using five concentration methods (Table 1): polyethylene glycol (PEG) precipitation (used in the Welsh and Northern Ireland surveillance programs), a modified PEG method with an initial elution with beef extract (30), ammonium sulfate (AS) precipitation (used for the national SARS-CoV-2 wastewater surveillance program in England), dead-end ultrafiltration using Amicon filters (used in the Scottish monitoring program), and tangential flow ultrafiltration with the InnovaPrep (IP) device, designed for wastewater testing. We also explored the trade-off between increasing sample volume and the efficiency of viral recovery. For verification, we used the two best-performing methods on neat (i.e., unspiked) wastewater samples.

**TABLE 1:** Experimental setup and sample number for spiking experiment^*a*^

Method code	Description	Water type	Water vol (mL)	Supernatant vol (mL)	Replicates (×)
A: PEG	Polyethylene glycol (PEG) precipitation	Distilled water	20 50 200	15 37.5 150	3 3 3
		Distilled water—spiked	20 50 200	15 37.5 150	3 2 3
		Wastewater	20 50 200	15 37.5 150	3 3 3
		Wastewater—spiked	20 50 200	15 37.5 150	3 3 3
B: BE-PEG	Elution with beef extract (BE)—PEG precipitation	Distilled water	20 50 200	15 37.5 150	3 3 3
		Distilled water—spiked	20 50 200	15 37.5 150	3 3 3
		Wastewater	20 50 200	15 37.5 150	3 3 3
		Wastewater—spiked	20 50 200	15 37.5 150	3 3 3
C: AS	Ammonium sulphate (AS) precipitation	Distilled water	20 50 200	15 37.5 150	3 3 3
		Distilled water—spiked	20 50 200	15 37.5 150	3 3 3
		Wastewater	20 50 200	15 37.5 150	3 3 3
		Wastewater—spiked	20 50 200	15 37.5 150	3 3 3
D: AM	Amicon (AM) filtration	Distilled water	20 50	15 20	3 3
		Distilled water—spiked	20 50	15 20	3 3
		Wastewater	20 50	15 20	3 3
		Wastewater—spiked	20 50	15 20	3 3
E: IP	InnovaPrep (IP) filtration	Distilled water—spiked	20 50	15 37.5	3 3
		Wastewater—spiked	20 50	15 37.5	3 3

a Water volume is the volume of the sample taken and centrifuged to clarify the samples from solid matter. Supernatant volume refers to the volume of the clarified solution concentrated.

## RESULTS

### Virus recovery in deionized water and wastewater: laboratory spiking experiment.

In this experiment, deionized and wastewater samples were spiked with SARS-CoV-2, influenza (flu) A/B, NoVGII, RoV, and MeV. Due to the overall high level of PMMoV, AdV, and crAssphage in wastewater, no spiking was performed for these viral targets. The nonspiked deionized water samples were negative for the target viruses, suggesting no cross-contamination. The unspiked wastewater samples were positive for SARS-CoV-2 and RoV; however, the levels were 1 to 2 orders of magnitude lower than those of the spiked virus concentrations and were deemed negligible during analysis.

The generalized linear model identified sample starting volume, concentration method, and water type as significant predictors of viral recovery (Table 2). The model coefficients reveal that sample starting volume was a significant negative correlate of viral recovery; the Amicon method had the highest mean recovery; and wastewater had reduced recovery over deionized water.

**TABLE 2:** Generalized linear model with gamma residuals (link = log) predicting recovery of 11 viruses in the liquid phase^*a*^

Variable	Model estimate
Intercept	3.759 (0.078)***
Vol	–0.007 (0.001)***
Method: AS	–1.245 (0.092)***
Method: BE-PEG	–0.368 (0.092)***
Method: IP	–1.55 (0.101)***
Method: PEG	–1.569 (0.094)***
Water type: WW	–0.848 (0.057)***
AIC^*b*^	4,748.24
R squared	0.597
Adjusted R squared	0.594

a Pellet methods were excluded from the model due to nonstandard starting volumes. The model results include the variable coefficient on a log scale indicating its effect (positive numbers indicating increased recovery), followed by the standard error in parentheses and a significance code (***, *P* < 0.001) rounded to three decimal places. The Amicon method and deionized water (DW) are predicted using the intercept.

b AIC, Akaike information criterion.

**FIG 1:**
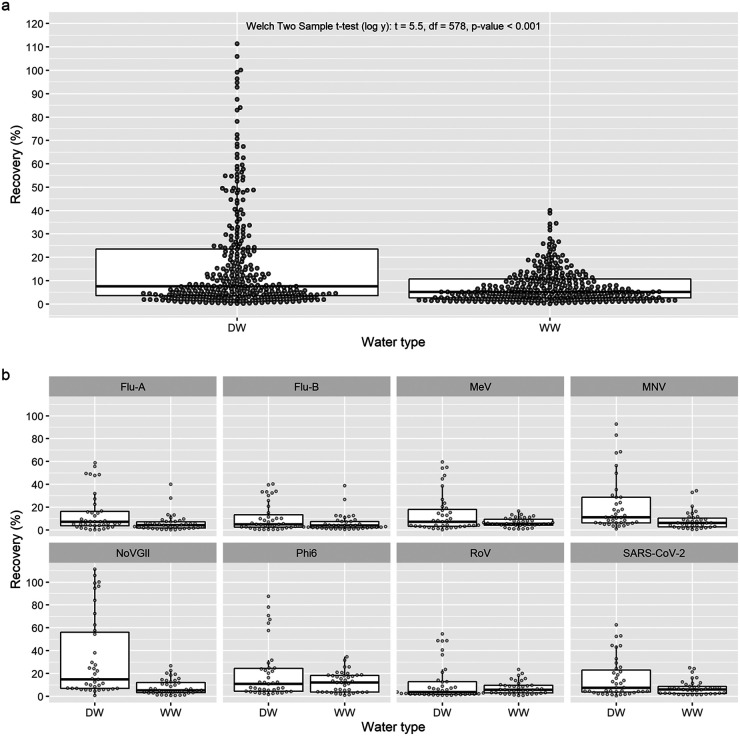
Greater recovery of spiked viruses, influenza A/B viruses (flu-A/B), measles virus (MeV), murine norovirus (MNV), SARS-CoV-2 (N1), norovirus GII (NoVGII), bacteriophage phi6 (Phi6), and rotavirus (RoV) in deionized water (DW) compared to wastewater (WW). Data derived from all concentration methods. (a) All spiked virus recovery results combined. (b) Recovery by individual virus. Boxes depict the 25th, 50th, and 75th percentile ranges after omitting outliers greater or less than ±1.5× the interquartile range (IQR), which is shown by the whiskers.

**(i) Greater recovery of spiked virus in deionized water compared to wastewater regardless of concentration method.** Deionized water had greater median recovery of spiked virus (7.64%) compared to wastewater (5.23%), which was found to be significant when comparing mean log_10_ transformed viral recovery values (Fig. 1a; Welch two-sample *t* test [log *y*]: *t* = 5.5, df = 578, *P* < 0.001). Greater recovery from deionized water over wastewater was seen in all viruses except for Phi6 and RoV (Fig. 1b). The highest recoveries (38 to 100%) were observed for all viruses when the Amicon ultrafiltration method was used on deionized water samples. These results suggest that wastewater contains other chemicals or materials which reduce the efficiency of the concentration and extraction steps or the qPCR amplification process.

**FIG 2:**
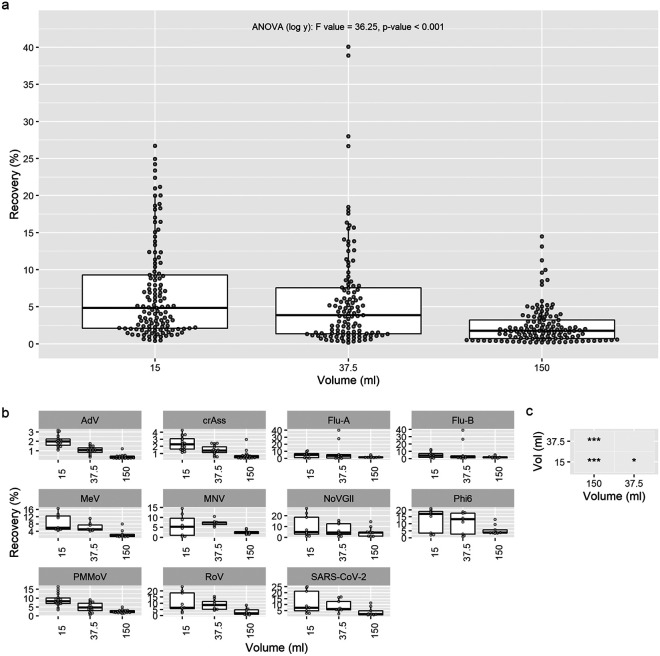
Recovery for human mastadenovirus (AdV), crAssphage (CrAss), influenza A/B virus (Flu-A/B), measles virus (MeV), murine norovirus (MNV), SARS-CoV-2 (N1), norovirus GII (NoVGII), bacteriophage phi6 (Phi6), pepper mild mottle virus (PMMoV), and rotavirus (RoV) as a function of starting volume of wastewater. Data derived from all concentration methods. (a) All recovery results combined. (b) Recovery separated by virus with a variable *y* scale (recovery percentage). (c) Existence of any significant differences in the tested volumes. (To analyze which volume is significantly better for viral recovery, panel c is to be analyzed in conjunction with panel ‘a and panel b). The *P* values (Holm adjustment method) of pairwise comparisons were calculated between extraction volumes with two sample *t* tests with pooled standard deviations (***, *P* < 0.001; **, *P* < 0.01; *, *P* < 0.05). Comparisons were made with an ANOVA after log_10_ transformation of recovery, followed by pairwise *t* tests; Pairwise comparisons found significant differences between all volumes with the Holm adjustment method (*P* < 0.05).

**(ii) Viral recovery improved with a reduced starting volume of wastewater.** The lowest starting volume (15 mL) had the greatest median viral recovery (4.85%), followed by 37.5 mL (3.84%) and then the largest volume (150 mL; 1.75%; Fig. 2a). The mean log_10_ transformed viral recovery was significantly different between groups (analysis of variance [ANOVA] *[log y]*: F value = 36.25, *P* < 0.001), as well as all pairwise comparisons (Fig. 2c; pairwise *t* tests with pooled standard deviation [SD]; *P* < 0.05; Holm adjustment method). The negative trend between volume and recovery was seen in the recovery of all individual viruses. Although MNV and RoV had higher median recovery for 37.5-mL samples compared to 15-mL aliquots (Fig. 2b), the difference was not significant (*P* > 0.05).

**FIG 3:**
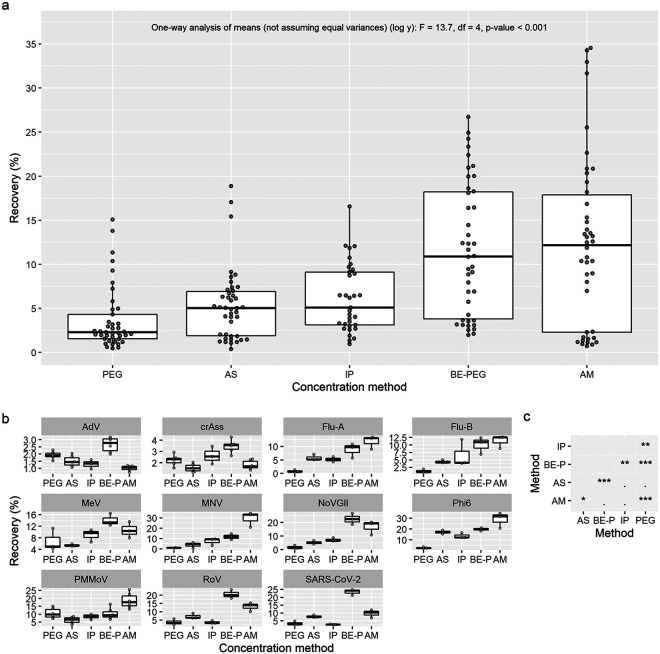
Influence of concentration methods on virus recovery from wastewater at a sample starting volume of 15 mL. (a) All recovery results with starting volumes of 15 mL combined. (b) Recovery separated by virus with a variable *y* scale. (c) Existence of any significant differences among concentration methods. (To analyze which volume is significantly better for viral recovery, panel c is to be analyzed in conjunction with panel a and panel b). The *P* values of pairwise comparisons of method recovery were calculated using *t* tests without pooled standard deviations (***, *P* < 0.001; **, *P* < 0.01; *, *P* < 0.05; -., *P* > 0.05; *P* value Holm adjustment method). Comparisons were made with an ANOVA after log_10_ transformation of recovery, followed by pairwise *t* tests (c); BE-PEG and Amicon methods had the highest median recovery, but due to Amicon method’s greater variance, pairwise comparisons with IP (third-highest median recovery) were only significantly different for BE-PEG (*P* < 0.05; panel c).

**(iii) BE-PEG and Amicon concentration methods have the greatest viral recovery.** Different concentration methods had a major effect on viral recovery (Fig. 3); Amicon and modified PEG (BE-PEG) methods had the highest median recovery (12.2% and 10.9%, respectively), followed by IP (5.1%) and then AS (5.0%) and PEG methods (2.3%). The variance of the Amicon method was significantly different from all other methods (*P* < 0.05), so a Welch ANOVA was selected (one-way analysis of means not assuming equal variances), which found significant differences between the method’s mean log_10_ transformed recovery (one-way analysis of means [not assuming equal variances] [log *y*]: F = 13.7, df = 4, *P* < 0.001). *Post hoc* pairwise comparisons with *t* tests found that the Amicon method’s viral recovery was not significantly different from that of the IP method due to the greater variance observed in the Amicon results, whereas BE-PEG method recoveries were significantly different from all other methods except Amicon (Fig. 3c; *P* value Holm adjustment method). The performance of the Amicon method compared to other methods varied highly between viruses, performing well for flu, MNV, and PMMoV while poorly for AdV and CrAss (Fig. 3b). The BE-PEG method, on the other hand, was consistently better than other methods (excluding Amicon) for individual virus recovery. These results suggest the BE-PEG method provides the greatest and most consistent viral recovery.

**FIG 4:**
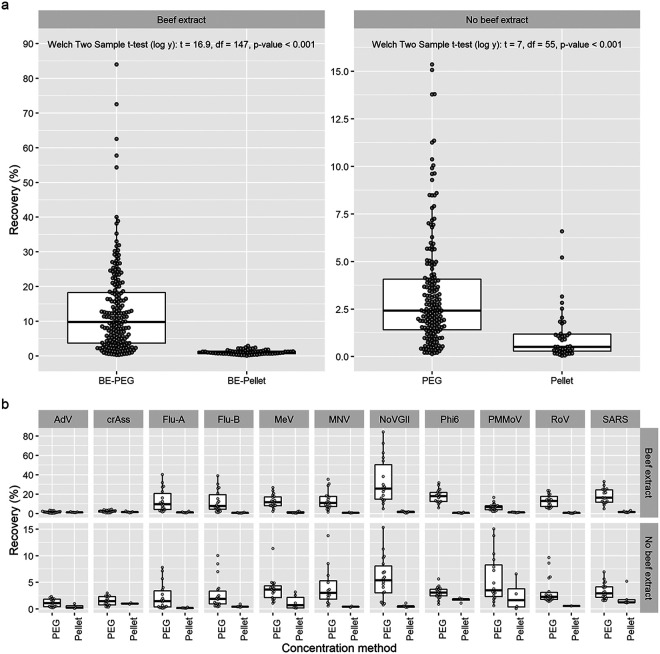
Viral recovery in the pellet from the first centrifugation step (10,000 × *g*, 10 min, 4°C) in the viral extraction procedure. The sample solid fraction (pellet) has significantly lower viral recovery than the concentrated sample; thus, removal via centrifugation will likely increase the median viral recovery of a concentrated sample. (a) All recovery results with starting volumes of 50 mL combined. (b) Recovery separated by virus with a variable *y* scale. Comparisons were made with a Welch two-sample *t* test after log_10_ transformation of recovery.

**(iv) Solid fraction may contain few virus particles.** The pellet recovered after the first centrifugation step (after spiking) had significantly lower viral recovery than the BE-PEG and PEG concentrates (Fig. 4a; (BE-PEG) Welch two-sample *t* test [log *y*]: *t* = 16.9, df = 147, *P* < 0.001; (PEG) Welch two-sample *t* test [log *y*]: *t* = 7, df = 55, *P* < 0.001). The pellet had consistently lower recovery of all individual viruses (Fig. 4b). These results suggest that a greater proportion of spike virus is suspended in the liquid of a sample compared to the solid fraction.

### Virus recovery in neat wastewater samples.

**(i) Amicon and BE-PEG concentration methods varied in recovery depending on the target virus.** The two best-performing wastewater concentration methods, the Amicon and BE-PEG methods, were tested on neat, unspiked wastewater samples collected at 13 wastewater treatment plants (Table 1). The 115 samples included grab and 24-h composite samples and showed great variations in physicochemical characteristics (Table 1).

We found significantly greater Phi6 (process control virus) recoveries when the BE-PEG method was used (4.51%) than for the Amicon method (0.77%). We did not find any influenza B, HIV, or hepatitis B/C viruses; however, crAssphage, SARS-CoV-2, flu A, EV, EVD68, NoV GI/GII, and measles virus were detected. The viruses detected sporadically (flu A: 0.88% detection rate with Amicon and 0% with BE-PEG; measles virus: 1.75% with Amicon and 2.63% with BE-PEG) were excluded from further analysis.

**FIG 5:**
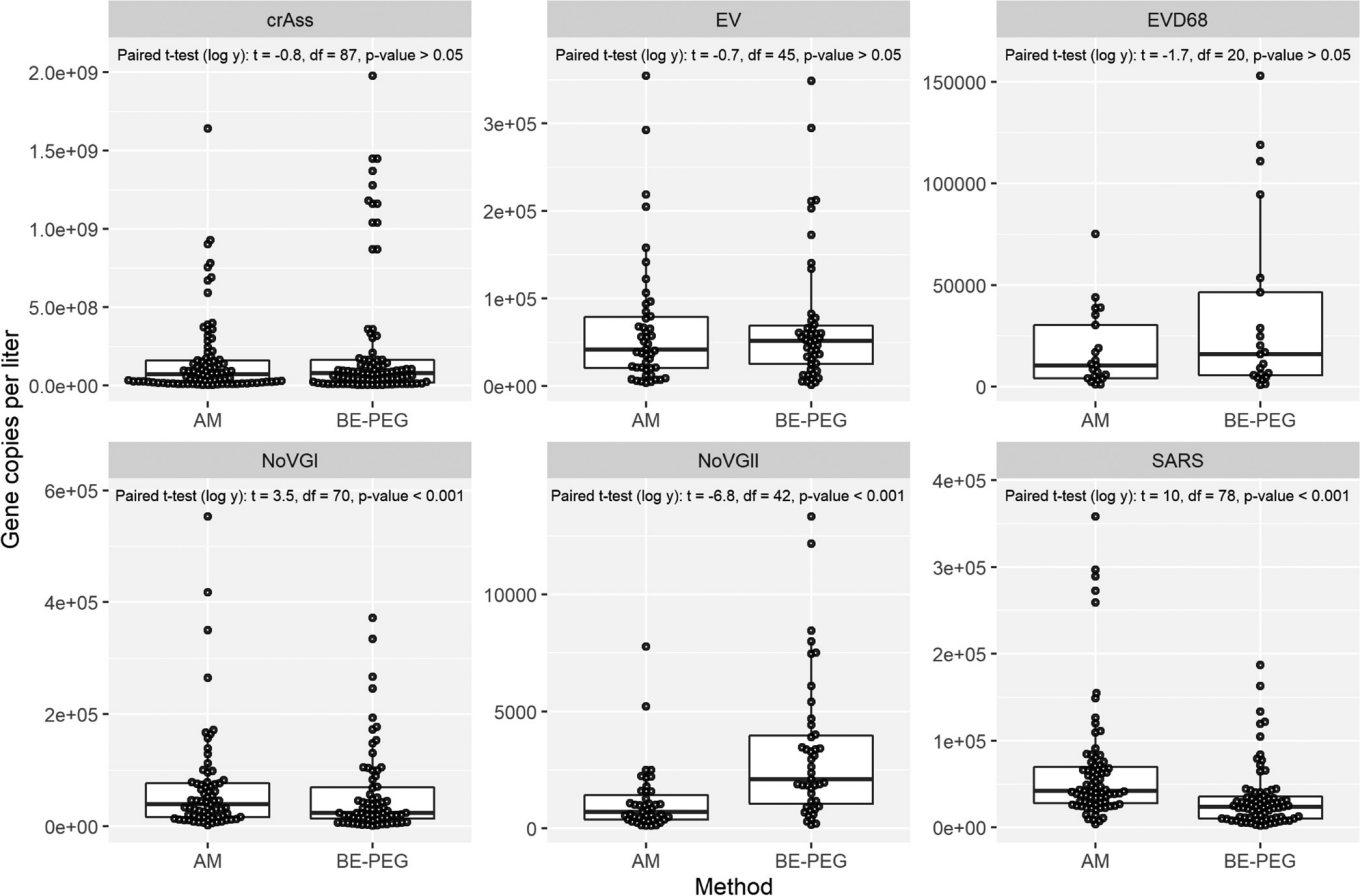
Comparison of viral recovery for Amicon and BE-PEG concentration methods tested on neat, unspiked wastewater samples collected at 13 wastewater treatment plants. Statistical comparisons were made using paired *t* tests after log transformation of the gene copies per liter. Recovery of crAss, enterovirus (EV), and enterovirus D68 (EVD68) could not be assumed to have differing means, while SARS-CoV-2 and norovirus GI (NoVGI) had significantly greater mean recovery with the Amicon method, and NoVGII had significantly greater mean recovery with the BE-PEG method.

The statistical analyses revealed that the BE-PEG and Amicon methods had various performances depending on the target virus (Fig. 5). Median gene copies (gc) per liter were similar between methods when targeting crAssphage (Amicon: 7 × 10^7^ gc/L; BE-PEG: 7.7 × 10^7^ gc/L), EV (Amicon: 4.2e × 10^4^ gc/L; BE-PEG: 5.2 × 10^4^ gc/L), and EVD68 (Amicon: 1.0 × 10^4^ gc/L; BE-PEG: 1.6 × 10^4^ gc/L), and mean log_10_ transformed recoveries were not found to differ significantly between methods (paired *t* test; *P* > 0.05). Conversely, the Amicon method recovered greater median concentrations when targeting SARS-CoV-2 (Amicon: 4.2 × 10^4^ gc/L; BE-PEG: 2.4 × 10^4^ gc/L) and NoVGI (Amicon: 3.9 × 10^4^ gc/L; BE-PEG: 2.4 × 10^4^ gc/L), while the BE-PEG method recovered greater median concentrations when targeting NoVGII compared to the Amicon method (Amicon: 7 × 10^2^ gc/L; BE-PEG: 2.1 × 10^3^ gc/L), differences that were found to be significant when comparing mean log_10_ transformed concentrations (paired *t* test; *P* < 0.001). These conflicting results suggest that neither method is consistently better than the other.

**(ii) The Amicon method performs comparatively better than BE-PEG as concentrations of ammonium increase.** The linear mixed effects model (lmm) analysis found that ammonium concentration had a significant positive effect (*P* < 0.01) on method performance, suggesting that the Amicon method is better suited for samples with high concentrations of ammonium inhibitors. However, none of the other chemistry variables had significantly different effects on the performance of the Amicon and BE-PEG methods (*P* > 0.05), and the intercept was not significant (see Fig. S10 in the supplemental material).

## DISCUSSION

In this study, we compared the performance of wastewater concentration methods currently used for COVID-19 monitoring in the United Kingdom (13) for the detection of different human viruses. Overall, the results suggested that all methods are suitable for routine viral surveillance; however, there were significant differences in their ability to recover viruses.

The first step of all methods we compared was the elimination of solid matter using centrifugation. This step is commonly used prior to virus concentration in wastewater samples, especially prior to ultrafiltration, as solid matter may cause membrane clogging (23, 31). However, virus particles may be attached to the solid matter in samples with high conductivity and/or with high levels of organic matter (32) and hence be excluded from virus concentration, resulting in low viral titers. In this study, we found that only a small proportion of the viruses attach to the solid particles in the pellet fraction, similar to our previous study where negligible amounts of SARS-CoV-2 RNA was recovered in the pellets of wastewater from six treatment plants in the United Kingdom (11). However, other studies suggest that up to 23% of SARS-CoV-2 can be recovered from the pellet fraction (33, 34). The contradictory findings suggest that there may be substantial differences in the virus adsorbing capacity to solid matter in different wastewater samples. Therefore, the presence of virus in the solid fraction cannot be ruled out. We note that higher viral loads may be found in the primary settling tank at wastewater treatment plants, as the concentration of solids is much greater (>100-fold) than in influent wastewater.

We found consistently higher viral recoveries from deionized water than in wastewater, regardless of the concentration method applied. Furthermore, increased wastewater volumes also had a negative impact on the recoveries of all viruses; however, the PEG method was less affected by volume increase than the AS, BE-PEG, Amicon, and IP methods. This suggests that organic matter (e.g., polysaccharides, ribonucleases) and other inhibitors of extraction and PCR coconcentrate with viruses (35). The Phi6 bacteriophage (used as a process control) also showed high variations in the neat/raw wastewater samples. Similar observations were made in other studies using transmissible gastroenteritis coronavirus (36), F-specific RNA (FRNA) bacteriophages (9, 37) and mengovirus (38) as a process control. Due to inconsistent wastewater matrices having different effects on viral recovery, a representative process control virus should always be used for virus concentration efficacy evaluation and control (39). In future WBE programs, it is likely that a multivirus process control will be needed to cover the range of viral types targeted.

It is important to note that we observed differences in the recoveries of different viruses, which may be due to differences in the structure, shape, size, or genetic material and their culturing, inactivation, and degradation processes. Due to environmental factors (e.g., temperature, physical pressure), viruses may become inactivated, releasing the genetic material in the environment where the genome size and structure would affect viral stability. The effect of viral properties on recoveries in wastewater should be addressed in future studies.

We found significant differences in the performance and applicability of different sample process methods (Table 3). Overall, the Amicon ultrafiltration concentration method gave high viral recoveries; however, the Amicon method showed great variations between viruses and replicates. Similar recoveries were observed using centrifugation-based ultrafiltration for process control viruses, such as murine hepatitis virus (56.0% ± 32.3%) (34), MS2 bacteriophage (33.3 ± 15.6%) (40), human coronavirus OC43 strain, (24% ± 2%) (41), and SARS-CoV-2 (25.9% to 65.3%) (42). In our study, Amicon filtration was only suitable for low sample volumes, up to 20 mL. However, the sample volume may be increased to up to 60 mL if samples are filtered through 0.45- and/or 0.2-μm filters to eliminate debris (41, 43). Another disadvantage of Amicon filtration was that the volume of the resulting concentrate was also inconsistent, and it may be too high for certain RNA/DNA extraction methods. Furthermore, depending on the sample characteristics, ultrafiltration times also varied from sample to sample, resulting in long centrifugation times, which may have a negative effect on virus integrity and recovery.

**TABLE 3:** Comparison and ranking of the methods used for virus concentration in wastewater^*a*^

Method	Viral recovery (%)	Sample vol (mL)	Concn factor (×)	Effective vol (mL)	Benchtop time/sample (min)	Overall time/sample (h)	Cost/sample (£)
PEG	3.3	150	1500	6	50	18	<1
BE-PEG	13.2	150	1500	6	60	18	<1
AS	6.2	150	1500	6	40	3	<1
IP	6.2	37.5	375	1.5	20	1	25
Amicon	14.1	15	150	0.6	30	1–2	8

a The effective volume is the proportional volume of the original wastewater sample assayed by RT-qPCR (4 μL/reaction).

The IP ultrafiltration method enabled rapid viral concentration from up to 50 mL of wastewater; however, the viral recoveries were lower than those of the Amicon ultrafiltration. A previous study also found that centrifugation-based ultrafiltration outperforms the IP method for the recovery of murine hepatitis virus (33). Similarly, high recoveries were observed when process control viruses, such as bovine coronavirus (16.8% to 53.2%) and MS2 bacteriophage (53.6%) were concentrated (44). However, another study showed significantly lower recoveries for bovine coronavirus (5.5%) and bovine respiratory syncytial virus (7.6%) (45) when using IP concentration, suggesting that the water type and composition affect viral recovery. The IP method was also useful as a secondary concentration method for a large volume of wastewater for the human coronavirus OC43 strain, resulting in 48% ± 2% recoveries (21); however, it failed to concentrate poliovirus (0.32% recovery) (46). Due to the simplicity and rapid process, IP methods have been used for SARS-CoV-2 surveillance in wastewater in the United States (12, 45). Nonetheless, the IP consumables are more expensive than the reagents and consumables used in the other methods, and hence the method may not be feasible for routine mass testing.

From the precipitation-based concentration methods, the BE-PEG method gave the best viral recoveries, similar to those for Amicon filtration, whereas the PEG and AS precipitations were less efficient for viral recovery. PEG precipitation has been widely used for viral recovery from large volumes of water and wastewater samples for decades (24, 25, 31, 47), and the addition of beef extract-sodium nitrate elution has also been shown to enhance viral recovery (30, 48). PEG induces the precipitation of viruses from the solution by reducing the solubility of proteins, while the beef extract enhances the precipitation by allowing the viral particles to bind it, which explains the recovery superiority of BE-PEG over PEG precipitation (49). The sodium nitrate added increases conductivity and hence assists in the detachment of virus particles from organic matter (50). Studies also showed that precipitation-based methods outperform IP concentration for the recovery of poliovirus, crAssphage, and SARS-CoV-2, suggesting that this process is robust and more resilient to organic matter than ultrafiltration (12, 51), whereas others found that PEG precipitation performs similarly to Amicon ultrafiltration, with both methods resulting in approximately 40% SARS-CoV02 recoveries (29). The precipitation-based methods are very easy to perform, and the associated reagents are inexpensive and usually available in bulk quantities. The bench time required for these methods is short, and many samples can be handled at once; however, the incubation times, especially for the PEG methods, significantly increase the overall processing time (29). Another disadvantage of precipitation-based methods is the potential resuspension of the pellet concentrate after centrifugation, and hence this step should be performed without delays.

We also used the two best-performing methods, Amicon and BE-PEG, for mass testing of wastewater samples. Both methods successfully detected most of the target viral sequences, except for influenza B virus and hepatitis B/C viruses and HIV. The influenza B infection rates were very low in the study area during wastewater sampling, which explains the lack of viruses in the samples. However, for viral genomes associated with the viral families *Flaviviridae*, *Hepadnavididae*, and *Retroviridae* in wastewater using sequencing-based approaches (52–55), these viruses degrade rapidly in the environment, and hence we were not able to identify hepatitis B/C viruses and HIV in the samples. The higher viral detection rates observed using the BE-PEG method (150 mL sample volume) compared to the Amicon method (15 mL sample volume) suggested that the precipitation of higher sample volumes enables the detection of rare viral targets. The quantitative analysis found no significant differences in the measured concentrations, suggesting that both methods are suitable for abundant targets. The only notable difference in the two methods was their performance at high ammonium levels, suggesting that the Amicon method may be preferable when catchments have high concentrations of ammonium, including those with high levels of agricultural runoff entering the sewage network.

**Conclusions and future work.** We found that all five methods are suitable for wastewater testing for virus quantification using quantitative PCR (qPCR), with the Amicon ultrafiltration and beef extract elution-PEG precipitation methods performing the best. However, great variations between viruses and replicates in viral recoveries were observed using the Amicon method. The Amicon method is suitable for small sample volumes with high ammonium levels and therefore should be used when abundant viruses are quantified, whereas the precipitation method may be used for early detection when levels are low and for rare pathogens. We also found that the composition and volume of wastewater significantly affects virus recovery, and therefore sample volume should be carefully chosen. Future work may include pretreatment procedures (e.g., increased salinity) to enhance viral recovery from large volumes of sewage and the better understanding of the effects of virus characteristics on viral recoveries.

## MATERIALS AND METHODS

### Wastewater collection and chemical analysis.

For the spiking experiment, raw sewage was collected on 5 November 2021 at wastewater treatment plant 1 (WWTP1). The wastewater was stored at 4°C until use. For surveillance, raw sewage samples were collected at 13 treatment plants in England four times a week over a 2-week period from 12 November to 25 November 2021 (*n *= 115; Table 4). The samples were transported to the laboratory, chilled overnight, and stored at 4°C. The sample process started within 24 h after the samples were taken. The sample pH, turbidity, electrical conductivity, and ammonium and orthophosphate ion concentrations were measured as described previously (11).

**TABLE 4:** Wastewater sampling sites and physicochemical properties of the wastewater samples analyzed in this study^*a*^

Site	Type	*n*	pH	Turbidity (NTU)	Conductivity (μS/cm)	Ammonium (mg/L)	Phosphate (mg/L)
WWTP1	Grab	1	7.12	10.6	2145	5.0	2.06
WWTP2	Grab/comp	8	7.45 ± 0.05	53.5 ± 12.3	889 ± 126	25.3 ± 7.9	2.15 ± 0.51
WWTP3	Grab	1	7.61	202.0	1,212	5.1	3.09
WWTP4	Grab/comp	9	7.52 ± 0.06	95.1 ± 18.7	1,644 ± 260	30.0 ± 5.0	2.90 ± 0.33
WWTP5	Comp	10	7.57 ± 0.04	101.3 ± 8.7	1,236 ± 91	45.2 ± 5.9	2.56 ± 0.23
WWTP6	Grab/comp	9	7.59 ± 0.09	64.3 ± 11.3	1,225 ± 264	38.9 ± 16.4	2.68 ± 0.64
WWTP7	Grab/comp	9	7.57 ± 0.07	103.6 ± 15.7	1,208 ± 98	26.0 ± 6.0	2.39 ± 0.31
WWTP8	Comp	9	7.37 ± 0.06	134.3 ± 20.5	1,076 ± 251	24.8 ± 7.8	2.11 ± 0.51
WWTP9	Grab/comp	9	7.46 ± 0.08	53.1 ± 11.9	1,042 ± 105	28.3 ± 6.3	3.47 ± 0.36
WWTP10	Grab/comp	7	7.51 ± 0.05	117.3 ± 21.4	1,175 ± 109	32.3 ± 8.8	3.13 ± 0.42
WWTP11	Grab/comp	9	7.60 ± 0.07	103.8 ± 30.9	1,458 ± 150	33.8 ± 4.9	2.61 ± 0.26
WWTP12	Grab/comp	9	7.56 ± 0.06	119.3 ± 13.3	2,069 ± 801	35.1 ± 7.8	3.04 ± 0.33
WWTP13	Grab/comp	8	7.39 ± 0.10	61.3 ± 10.1	1,686 ± 249	26.1 ± 6.5	2.40 ± 0.53
WWTP14	Grab/comp	17	7.62 ± 0.04	102.8 ± 19.3	1,089 ± 117	37.8 ± 4.6	2.92 ± 0.43

a Comp, 24-h composite sample. Values represent means ± standard error of the mean (SEM).

### Virus spiking.

Virus spiking was performed in a biosafety level 2/containment level 2 (BSL2/CL2) laboratory using a class II biosafety cabinet. Sample bottles were disinfected using 70% industrial methylated spirit (IMS) prior to removal from the cabinet.

For spiking, we used heat-inactivated SARS-CoV-2 (kindly provided by Andrew Weightman, Cardiff University), inactivated influenza A/California/07/2009 (H1N1), B/Lee/40, rotavirus SA11 (RoV) cultures (kindly provided by Eleanor Gaunt, University of Edinburgh), norovirus GII (NoVGII) in diluted and filtered fecal matter from a patient with confirmed norovirus infection (kindly provided by Lydia Drumwright, University of Cambridge), and measles virus (MeV) in the form of a vaccine (VWR International, USA). We also used non-human viruses which are commonly used as process controls, namely, Phi6 bacteriophage and murine norovirus (MNV), which we cultured in-house as described in Kevill et al. (51).

Wastewater collected from WWTP1 (Table 1) and deionized water samples (3 L each) were spiked with each virus to reach a final concentration of approximately 104 to 105 genome copies (gc)/mL per virus type. The same volumes of neat (i.e., unspiked) deionized water and wastewater samples were also prepared. A 0.5-mL aliquot of each water type (deionized water, deionized water plus viral spikes, wastewater, wastewater plus viral spikes) was saved in triplicate for direct RNA/DNA extraction to determine the concentration of each virus in the samples. The water samples were mixed by shaking and then aliquoted to 20, 50, and 200 mL for each method (Table 1). Overall, 143 samples were generated and processed as described below.

The rest of the wastewater samples collected at 13 locations in England were spiked only with Phi6 bacteriophage as a process control to estimate virus recovery.

### Wastewater concentrations.

All wastewater concentration processes were performed in a BSL2/CL2 laboratory using a class II biosafety cabinet. Sample bottles were disinfected using 70% IMS prior to removal from the cabinet.

**Method A: polyethylene glycol precipitation (PEG method).** The PEG precipitation method described previously for SARS-CoV-2 detection in wastewater (56) was used with small modifications. The samples were centrifuged at 10,000 × *g* at 4°C for 10 min to clarify the solutions. The pellets from the 50-mL samples were subject to direct RNA/DNA extraction, whereas the rest of the pellets were discarded. The pH of the supernatants (Table 1) was adjusted to 7 to 7.5 and then were mixed with PEG 8000 and NaCl to reach the final concentrations of 10% and 2%, respectively. The solutions were mixed by inverting several times and incubated at 4°C for 16 h. The mixture was then centrifuged at 10,000 × *g* at 4°C for 30 min. The resulting pellet was subject to RNA/DNA extraction.

**Method B: modified PEG precipitation (BE-PEG method).** This method implemented a beef extract elution to detach viruses from solid matter prior to PEG precipitation (30). The samples were mixed with Lab Lemco beef extract (Oxoid, USA) and sodium nitrate to reach the final concentrations of 3% and 2 M, respectively. The pH of the mixture was adjusted to 5.5, and then it was incubated at 50 rpm at room temperature for 30 min. The PEG precipitation protocol was then followed, as described above (Table 1). The first centrifugation pellets from the 50-mL samples were subjected to RNA/DNA extraction, whereas the rest of the pellets were discarded. This method was also used for the concentration of the surveillance samples (200 mL each).

**Method C: ammonium sulfate precipitation (AS method).** The AS precipitation method described previously for SARS-CoV-2 detection in wastewater was used (51). In brief, the samples were centrifuged at 10,000 × *g* at 4°C for 10 min to clarify the solutions. The supernatants (Table 1) were mixed with AS to reach a final concentration of 40%. The solutions were mixed by inverting them several times and were incubated at 4°C for 1 to 2 h. The mixture was then centrifuged at 10,000 × *g* at 4°C for 30 min. The resulting pellet was subjected to RNA/DNA extraction.

**Method D: ultrafiltration using the Amicon Ultra centrifugal filters (Amicon method).** The samples were centrifuged at 4,000 × *g* at 4°C for 10 min to clarify the solutions. The pellets were discarded, whereas the supernatants (Table 1) were transferred to 10-kDa Amicon Ultra-15 centrifugal filters (Merck Life Science UK Ltd., Watford, UK). The samples were centrifuged at 5,000 × *g* for 30 to 60 min to reach a final volume of 200 to 500 μL. The filtrates were discarded. This method was also used for the concentration of the surveillance samples (20 mL each).

**Method E: ultrafiltration using the InnovaPrep system (IP method).** The samples were mixed with 5% Tween 20 (Sigma-Aldrich, USA) to reach the final concentration of 0.05% Tween. The mixtures were then centrifuged at 10,000 × *g* at 4°C for 10 min to clarify the solutions. The pellets were discarded, whereas the supernatants (Table 1) were filtered using the InnovaPrep (IP) concentrating pipette with 0.05-μm polysulfone (PS) hollow fiber filter tips (CP Select, USA). The tips were changed between samples. Samples were eluted in 25 mM Tris elution fluid (CP Select, USA), which contains 0.075% Tween 20.

### Viral RNA/DNA extraction.

Viral RNA/DNA of both concentrated and unconcentrated samples were extracted using the NucliSens extraction system (bioMérieux, France) on a Kingfisher 96 Flex system (Thermo Scientific, USA) as described previously (51, 56). In brief, the samples were mixed and incubated with NucliSens lysis buffer for 10 min, followed by the addition of NucliSens magnetic silica beads with a 10-min incubation to allow the viral nucleic acids to bind to the beads. The binding was followed by washing steps using NucliSens wash buffers 1 to 3 and a final elution of RNA/DNA in NucliSens wash buffer 3. The final volume of the eluent was 100 μL.

### Viral RNA/DNA quantification.

The viral RNA/DNA were quantified with reverse transcriptase quantitative PCR (RT-qPCR) (RNA targets) and with qPCR (DNA targets). All qPCRs were performed using a QuantStudio Flex 6 real-time PCR machine (Applied Biosystems, Inc., USA). The primers and probes for the target viruses have been used and validated previously (51, 56). Primers, probes, standards, and reaction conditions are detailed in Table S1. For quantification, a dilution series of DNA/RNA standards incorporating the target sequence was used. Each reaction plate contained multiple nontemplate controls, which were negative throughout the study, suggesting no cross-contamination. Assay limit of detection and limit of quantification values and RT-qPCR quality control data are summarized in Table S2.

For all samples, the RNA targets were quantified with probe-based assays using the TaqMan viral 1-step RT-qPCR master mix (Applied Biosystems, Inc., USA) with 1 μg bovine serum albumin (BSA) in the reaction mixes. Additionally, 16 nmol MgSO_4_ was added to the reaction mix for the SARS-CoV-2, Phi6, and influenza targets. Duplex RT-qPCR assays were used for the SARS-CoV-2 N1 gene fragment and the Phi6 phage (51) for influenza A and B (57) and for NoVGII and MNV (58), respectively. Singleplex assays were used for measles virus (59) and for rotavirus (RoV) with a commercial primer and probe mix (Primerdesign, UK). Adenovirus and crAssphage were quantified using the QuantiFast SYBR and the QuantiNova Probe qPCR reagents, respectively, with 1 μg BSA in the reaction mix, as described previously (30, 60).

The neat, unspiked samples from the surveillance study were also tested for human immunodeficiency virus (HIV) and hepatitis B and C viruses using a commercial triplex assay following the manufacturer’s protocols (Primerdesign, UK). Additionally, a duplex assay was used for the quantification of enteroviruses (EV) (Public Health Wales, personal communication) and enterovirus D68 (EVD68) (61), and a singleplex assay was used for norovirus GI (NoVGI) (58). When the Phi6 control recovery was <0.1%, the quantification was repeated with 2 μL sample/reaction; however, the reduced volumes did not affect recovery rates, suggesting little inhibition.

### Data analysis.

The initial qPCR data analysis and quality control were performed using the QuantStudio Flex 6 real-time PCR software v1.7.1 (Applied Biosystems, Inc., USA). The viral concentrations were expressed as gc/μL RNA/DNA extract. The viral concentrations (gc/L) in wastewater determined by concentration methods were calculated as

(1) $$\frac{\text{concentration of the nucleic acid extract}\;\times \;\text{extract volume}}{\text{volume of raw wastewater processed}}\;\times 1,000$$

The viral concentrations (gc/L) in the original unconcentrated samples were calculated as

(2) $$\frac{\text{concentration of the nucleic acid extract}\;\times \;\text{extract volume}}{\text{volume of sample extracted}}\;\times 1,000$$

Virus recoveries were calculated as

(3) $$\frac{\text{concentration of the concentrated samples}}{\text{concentration of the unconcentrated samples}}\;\times 100\%$$

Subsequent data analysis and statistical tests were carried out in R (62). For the samples from the laboratory spiking experiment, sample starting volume (15 mL, 37.5 mL, and 150 mL), concentration method (PEG, AS, IP, BE-PEG, and Amicon), and water type (wastewater [WW], and deionized water [DW]) were selected as factors and covariates of viral recovery. The selected features were assessed in combination as predictors of viral recovery using a generalized linear model (glm) with the response variable modeled as a gamma distribution with a logarithmic link function following an assessment of the right skewed response variable distribution (equation 4; see Fig. S1 for residual plots).

(4) $$\mathrm{Recover}y_{i}=\exp[\alpha_{0},+\;\alpha_{1}\;\mathrm{Volum}e_{i}\;+\;\alpha_{2}\;\mathrm{Metho}d_{i}\;+\;\alpha_{3}\;\mathrm{Water}\;\mathrm{typ}e_{i}+\;\epsilon ]$$

where *Recovery_i_* is the individual value of *N* total observations in the response variable and *i* relates to the individual observations of each variable. exp[…] indicates an exponential function of the product within the square brackets. *α_0_* is the intercept, *α*_1_,…*α*_3_ are the fixed-effects coefficient estimates, and *ϵ* is the error between the model prediction and observation *i* of the response variable.

Following the glm, comparisons of individual features were visualized, and statistical tests were performed. For statistical tests, the recovery percentile was log_10_ transformed to meet assumptions of a Gaussian distribution (see Fig. S2 to S6 for quantile-quantile [qq] plots). Equality of variances were tested with F tests. Statistical comparisons of features with two levels and nonequal variance were made with Welch two sample *t* tests. Comparisons with three or more levels with equal variance were made with a one-way ANOVA, followed by pairwise two-sample *t* tests with pooled standard deviations (SD), adjusting *P* values with the Holm-Bonferroni (H-B) method (63). Comparisons with three or more levels and nonequal variance were made with a Welch ANOVA (one-way comparison of means), followed by pairwise two-sample *t* tests without pooled SD, adjusting *P* values with the H-B method. Paired tests were not selected due to missing data created by removal of undetermined results and sample removal during qPCR quality control. Water type was compared with only spiked viruses due to the lack of naturally present virus in deionized water. Starting volume was compared using only wastewater to simulate typical interactions seen in wastewater projects and not using the Amicon or IP methods due to differing volumes. Method comparison was made using 15-mL wastewater sample volumes which were used in all concentration methods, including the Amicon and IP methods.

For the neat wastewater samples, viral recoveries with the Amicon and BE-PEG methods were compared for six viruses (crAssphage, EV, EVD68, SARS-CoV-2, NoVGI, and NoVGII). The recovery in gene copies per liter was log_10_ transformed to meet assumptions for parametric analysis, and then statistical comparisons were made using paired *t* tests. Residual qq plots can be found in Fig. S7. Concentrations of ammonium, electrical conductivity, and orthophosphate have been shown to correlate with human populations and thus relate to flow over short periods of time flow (13). Therefore, the variability in flow had to be removed to assess the comparative effect of water chemistry variables on each concentration method. To remove this variability, the viral concentrations (gc/L) recovered by the Amicon method were divided by the viral concentrations recovered by the BE-PEG method. As each sample was paired, both sample methods were influenced by the same variability in flow, which was removed through this division. A linear mixed effects model (lmm) was then used to assess the comparative effect of wastewater chemistry and quality variables (turbidity, pH, orthophosphate, electrical conductivity, and ammonium) on the proportion of each concentration method’s viral recovery, utilizing random effects for the target virus to borrow strength in a combined assessment of all viruses (equation 6). Prior to the model being fit, the dependent variable was power transformed using the Box-Cox method (equations 5 and 6) (64), with lambda (λ) selected by maximizing the log-likelihood of a multiple linear regression with all predictors used in the final linear mixed effects model. The residual plots of the final model can be found in Fig. S8.

(5) $$y(\lambda )=\{\frac{y^{\lambda }\;-\;1}{\lambda },\text{if}\;\lambda \;\neq 0;\;\log\;y,\;\text{if}\;\lambda =0\}$$

where *y* is the variable to be power transformed and *λ* is selected through maximizing the log-likelihood of a multiple linear regression with all predictors used in the final linear mixed effects model (equation 6).

(6) $$y{\left(\lambda \right)}_{\mathrm{ij}}\;=\;\beta_{0}\;+\;\beta_{1}\;\mathrm{Turbidit}y_{\mathrm{ij}}\;+\;\beta_{2}\;pH_{\mathrm{ij}}\;+\;\beta_{3}\;\mathrm{Orthophosphat}e_{\mathrm{ij}}\;+\;\beta_{4}\;\mathrm{Conductivit}y_{\mathrm{ij}}+\beta_{5}\;\mathrm{Ammoniu}m_{\mathrm{ij}}\;+\;\gamma_{j}\;\mathrm{Viru}s_{\mathrm{ij}}\;+\;\varepsilon$$

where *y*(*λ*)*_ij_* is the individual value of *N* total observations in the response variable after power transformation. *j* relates to group in the factor variable modeled with random effects, and the maximum *j* is equal to the number of levels in the target virus variable; *β*_0_ is the intercept, and *β*_1_,…,*β*_5_ are the fixed-effect coefficient estimates; *γ_j_* is the random-effect coefficients in group *j*; and *ε* is the error between the model prediction and observation *i* in group *j* (model parameters were fit independently of equation 4).

### Data availability.

The full script and data are provided in a dedicated repository (https://github.com/CameronPellett/Spiked-virus-concentration-Bangor and https://github.com/CameronPellett/Chem-Con-Bangor-Bath).
