# Supplementary material for: Comparative Assessment of Filtration- and Precipitation-Based Methods for the Concentration of SARS-CoV-2 and Other Viruses from Wastewater
Source: Microbiol Spectr. 2022 Aug 11;10(4):e01102-22. doi: 10.1128/spectrum.01102-22 (PMC9430619; doi:10.1128/spectrum.01102-22)
Supplement: Supplemental file 1 — Supplemental material. Download spectrum.01102-22-s0001.pdf, PDF file, 0.8 MB [file spectrum.01102-22-s0001.pdf]

Supplementary material

for

Comparative assessment of filtration- and precipitation-based methods for the concentration of SARS-CoV-2 and other viruses in wastewater

Kata Farkas[a,b,*], Cameron Pellett[a], Natasha Alex-Sanders[a], Matthew T.P. Bridgman[a], Alexander Corbishley[c], Jasmine M.S. Grimsley[d], Barbara Kasprzyk-Hordern[e], Jessica L. Kevill[a], Igor Pântea[a], India S. Richardson-O'Neill[a], Kathryn Lambert-Slosarska[a], Nick Woodhall[a], Davey L. Jones[a,f]

[a]Centre for Environmental Biotechnology, School of Natural Sciences, Bangor University, Bangor, Gwynedd, LL57 2UW, UK

[b]School of Ocean Sciences, Bangor University, Menai Bridge, Anglesey, LL59 5AB, UK

[c]Royal (Dick) School of Veterinary Studies and The Roslin Institute, University of Edinburgh, Easter Bush Campus, Roslin, EH25 9RG, UK

[d]UK Health Security Agency, Environmental Monitoring for Health Protection, Windsor House, Victoria Street, London SW1H 0TL, UK

[e]Department of Chemistry, University of Bath, Bath, BA2 7AY, UK

[f]Food Futures Institute, Murdoch University, 90 South Street, Murdoch, WA 6105, Australia

Table S1. qPCR standards primers, probes and conditions.

Assay type	Target virus	Target region (reference)	qPCR standards	Oligo type	Sequence and concentration/reaction	pmol in reaction mix	Reaction conditions
Duplex probe-based RT-qPCR	SARS-CoV-2	N1 gene fragment (*2019-Novel Coronavirus (2019-nCoV) Real-time rRT-PCR Panel Primers and Probes*, 2020)	In-house ssRNA standard (quantified using Qubit 2.0, Invitrogen, USA)	Forward primers	GACCCCAAAATCAGCGAAAT	10	Reverse transcription: 50°C, 30 min.<br>RT inactivation: 95°C, 20 s<br>45 cycles of: 95°C, 3 s and 60°C, 30 s.
				Reverse primers	TCTGGTTACTGCCAGTTGAATCTG	20	
				Probe	**FAM**-ACCCCGCATTACGTTTGGTGGACC-**MGB**	2.5	
	Phi 6 phage	*phi-6S_1* gene, coding P8 protein (Gendron et al., 2010)	In-house ssRNA standard (quantified using Qubit 2.0, Invitrogen, USA)	Forward primers	TGGCGGCGGTCAAGAGC	10	
				Reverse primers	GGATGATTCTCCAGAAGCTGCTG	20	
				Probe	**VIC**-CGGTCGTCGCAGGTCTGACACTCGC-**QSY**	2.5	
Duplex probe-based RT-qPCR	Influenza A	Matrix protein gene (CDC, 2021)	Synthetic influenza H1N1 (2009) RNA control (Twist Bioscience, USA)	Forward primers	CAAGACCAATCYTGTCACCTCTGAC<br>CAAGACCAATYCTGTCACCTYTGAC	10	
				Reverse primers	GCATTYTGGACAAAVCGTCTACG<br>GCATTTTGGATAAAGCGTCTACG	20	
				Probe	**FAM**-TGCAGTCCT/**ZEN**/CGCTCACTGGGCACG-**IABkFQ**	5	
	Influenza B	Non-structural protein gene (CDC, 2021)	Synthetic influenza B RNA control (Twist Bioscience, USA)	Forward primer	TCCTCAAYTCACTCTTCGAGCG	10	
				Reverse primer	CGGTGCTCTTGACCAAATTGG	20	
				Probe	**YakYel**-CCAATTCGA/**ZEN**/GCAGCTGAAACTGCGGTG-**IABkFQ**	5	
Duplex probe-based RT-qPCR	Norovirus GII	RdRp-ORF1 (*ISO/TS 15216-2:2019-Microbiology of food and animal feed — Horizontal method for determination of hepatitis A*	Plasmid DNA (quantified using Qubit 2.0, Invitrogen, USA) (Farkas et al., 2017)	Forward primer	ATGTTCAGRTGGATGAGRTTCTCWGA	10	
				Reverse primer	TCGACGCCATCTTCATTCACA	20	
				Probe	**FAM**-AGCACGTGGGAGGGCGATCG-**MGB**	5	

		virus and norovirus in food using real-time RT-PCR — Part 2: Method for qualitative detection, 2019)					
	Murine norovirus	ORF1–ORF2 junction region (Kitajima et al., 2010)	Plasmid DNA (quantified using Qubit 2.0, Invitrogen, USA)	Forward primer	CCGCAGGAACGCTCAGCAG	10	
				Reverse primer	GGYTGAATGGGGACGGCCTG	20	
				Probe	**DO**-ATGAGTGATGGCGCA-**DDQ**	5	
Duplex probe-based RT-qPCR	Enteroviruses	VP1 region (Public Health Wales)	Synthetic Enterovirus D68 RNA control (Twist Bioscience, USA)	Forward primer	GGTYGAAGAGYCTATTGAGC	10	
				Reverse primer	GCTCCGYIGTTRGGATTAGCCG	20	
				Probe	**HEX**-TCCTCCGGCCCCTG-**BHQ**	5	
	Enterovirus D68	5′ NTR region (Poelman et al., 2015)	Synthetic Enterovirus D68 RNA control (Twist Bioscience, USA)	Forward primer	TGTTCCCACGGTTGAAAACAA	10	
				Reverse primer	TGTCTAGCGTCTCATGGTTTTCAC	20	
				Probes	**FAM**-TCCGCTATAGTACTTCG-**MGB**<br>**FAM**-ACCCTATAGTACTTCG-**MGB**	5	
Singleplex probe-based RT-qPCR	Measles virus	Nucleoprotein gene (Hummel et al., 2006)	RNA extracted from vaccine (quantified against commercial standard (Primerdesign, UK)	Forward primer	TGGCATCTGAACTCGGTATCA	10	
				Reverse primer	TGTCCTCAGTAGTATGCATTGCAA	20	
				Probe	**FAM**-CCGAGGATGCAAGGCTTGTTTCAGA-**QSY**	5	
Singleplex probe-based RT-qPCR	Human rotavirus A	Commercial assay, Primerdesign, UK	Commercial assay, Primerdesign, UK	Forward primer	Not available		
				Reverse primer	Not available		
				Probe	Not available		

Singleplex probe-based RT-qPCR	Pepper mild mottle virus	Replication-associated protein gene (Haramoto et al., 2013)	Commercial ssDNA of the target sequence	Forward primer	GAGTGGTTTGACCTTAACGTTTGA	10	
				Reverse primer	TTGTCGGTTGCAATGCAAGT	20	
				Probe	**FAM**-CCTACCGAAGCAAATG-**MGB**	5	
Singleplex probe-based qPCR	CrAssphage	Putative DNA-binding protein (Stachler et al., 2017)	Plasmid DNA (quantified using Qubit 2.0, Invitrogen, USA)	Forward primer	CAGAAGTACAAACTCCTAAAAAACGTAGAG	5	Denaturation: 95°C, 5 min.<br>40 cycles of:<br>95°C, 15 s and 60°C, 1 min.
				Reverse primer	GATGACCAATAAACAAGCCATTAGC	10	
				Probe	**FAM**-AATAACGATTTACGTGATGTAAC-**TAMRA**	2.5	
Singleplex SYBR Green qPCR	Adenovirus	Hexon gene (van Maarseveen et al., 2010)	Plasmid DNA (quantified using Qubit 2.0, Invitrogen, USA)	Forward primer	CATGACTTTTGAGGTGGATC	10	Denaturation: 95°C, 5 min<br>40 cycles of:<br>95°C, 15 s and 55°C, 1 min.<br>Melting curve analysis:<br>95 °C, 15 s;<br>annealing 60°C, 1 min.<br>Dissociation: 60 °C to 95 °C (0.05 °C/s).<br>Melting peak (Tm) at<br>86.2 °C ± 0.3 °C.
				Reverse primer	CCGGCCGAGAAGGGTGTGCGCAGGTA	10	

Table S2. Limit of detection (LOD), limit of quantification (LOQ) values determined as genome copies (gc) per µl RNA/DNA extract, slope, R^2 and efficiency of the (RT-)qPCR assays. LOD and LOQ was determined as described in Farkas et al. (2017). N/A: not available.

**Target virus**	**LOD (gc/µl)**	**LOQ (gc/µl)**	**Slope**	**R^2**	**Efficiency (%)**
SARS-CoV-2 (N1)	0.917	12.597	-3.563 - -3.243	0.971-0.999	91.55-103.39
Phi 6 phage	N/A	N/A	-3. 579 - -3.164	0.969-0.999	90.27-107.03
Influenza A	1.825	7.12	-3.569 - -3.304	0.991-0.999	90.63-100.75
Influenza B	1.18	4.6	-3.550 – 3.361	0.995-0.999	91.28-98.38
Norovirus GI	N/A	N/A	-3.212 - -3.252	0.988-0.995	103.00 - 104.82
Norovirus GII	1.6	9.2	-3.51 - -3.554	0.994-0.996	91.14-92.69
Murine norovirus	N/A	N/A	-3.473	0.973	94.07
Measles virus	N/A	N/A	-3.107 - -3.196	0.979-0.994	105.52-109.83
Human rotavirus A	N/A	N/A	-3.251 - -3.547	0.995-0.998	91.38-103.03
Pepper mild mottle virus	N/A	N/A	-3.381	0.997	97.60
CrAssphage	2.305	12.895	-3.449 - -3.125	0.973-0.998	94.95-108.91
Adenovirus	N/A	N/A	-3.360 - -3.362	0.995-0.996	98.37-98.45

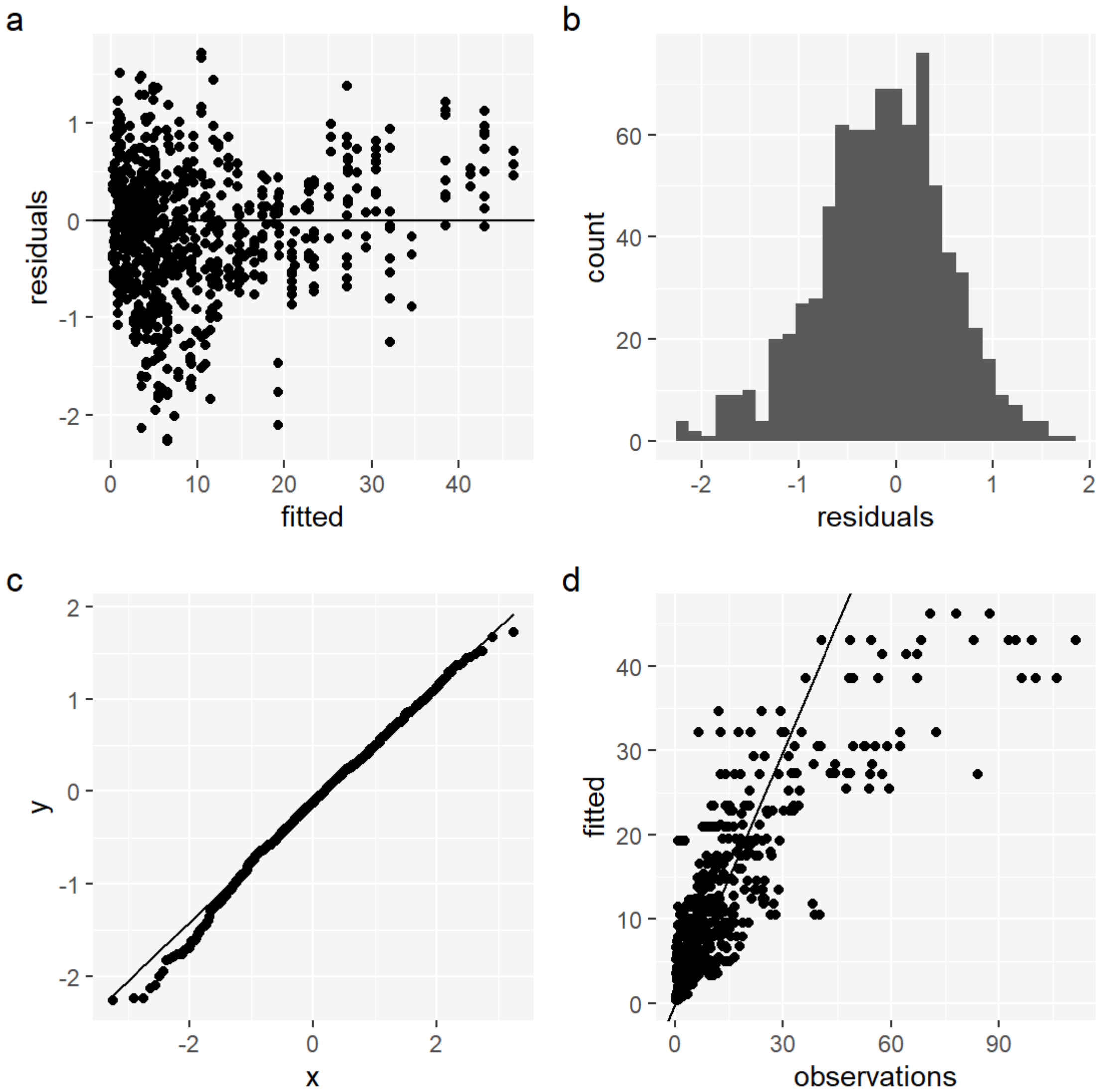

Figure S1: Residual plot of the generalised linear model fitted as a Gamma distribution with a logarithmic link function. Residual plots of (a) homoscedasticity, (b) histogram normality, (c) quantile-quantile normality, and (d) true observations against fitted values.

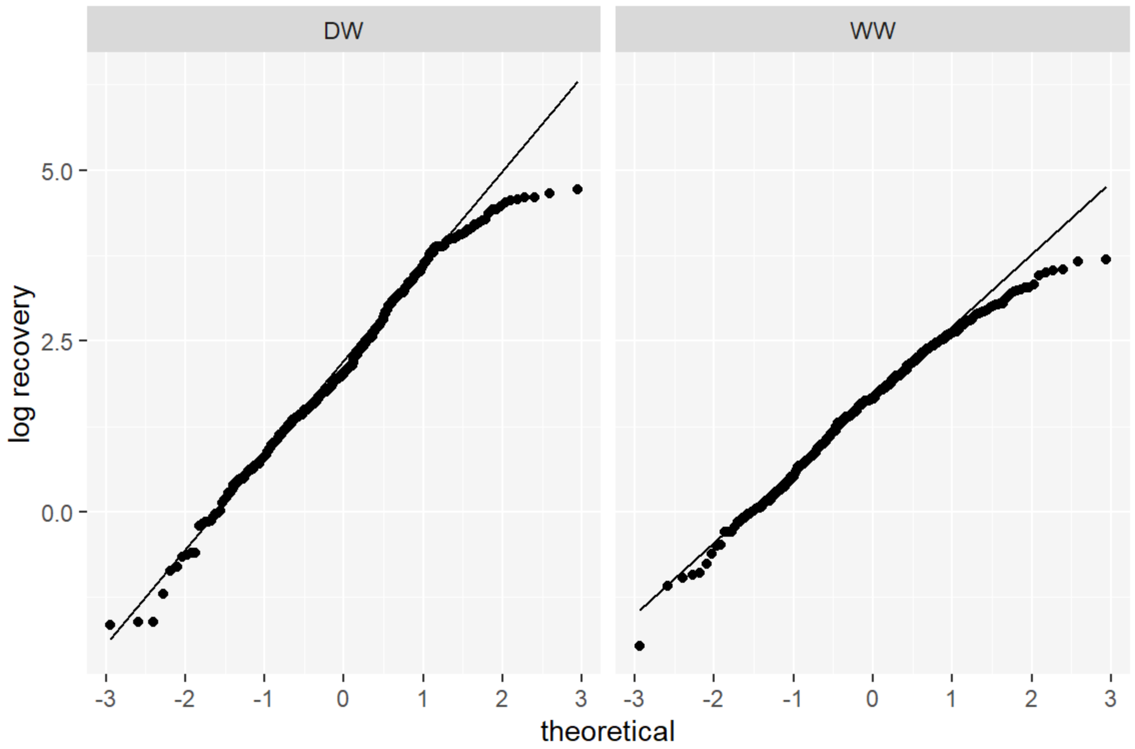

Figure S2: Quantile-quantile plot of $\log_{10}$ transformed virus recovery grouped by water type (De-ionised water DW and wastewater WW).

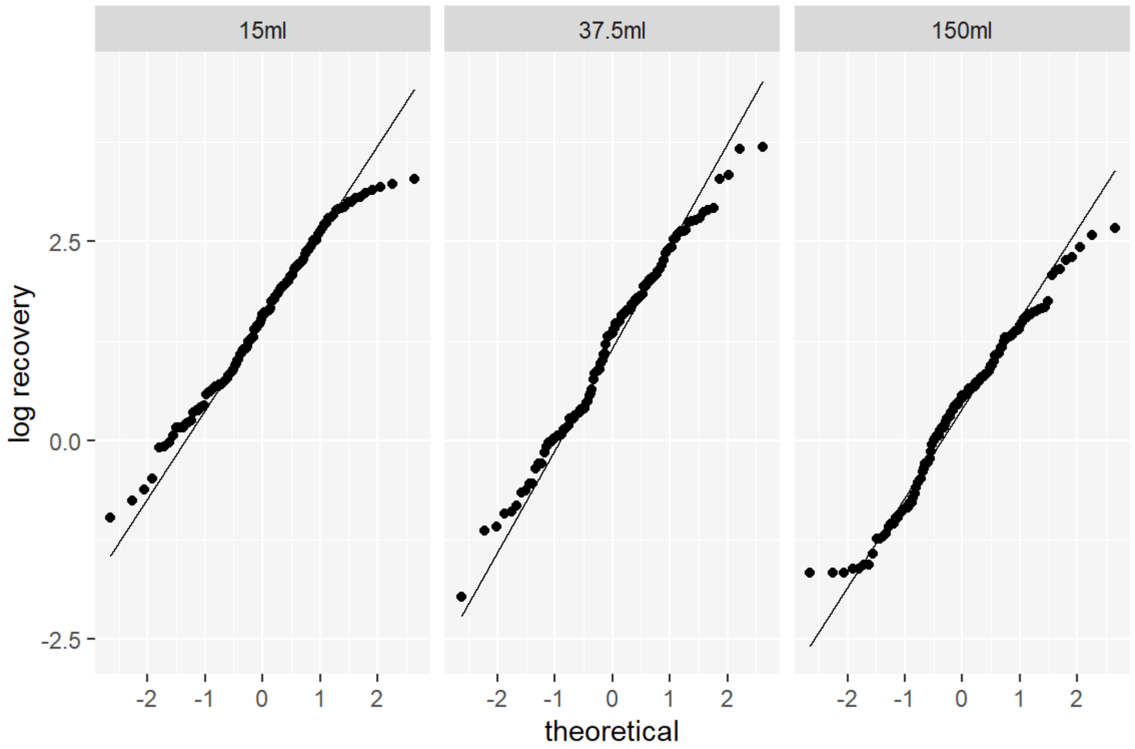

Figure S3: Quantile-quantile plot of $\log_{10}$ transformed virus recovery grouped starting volume of wastewater.

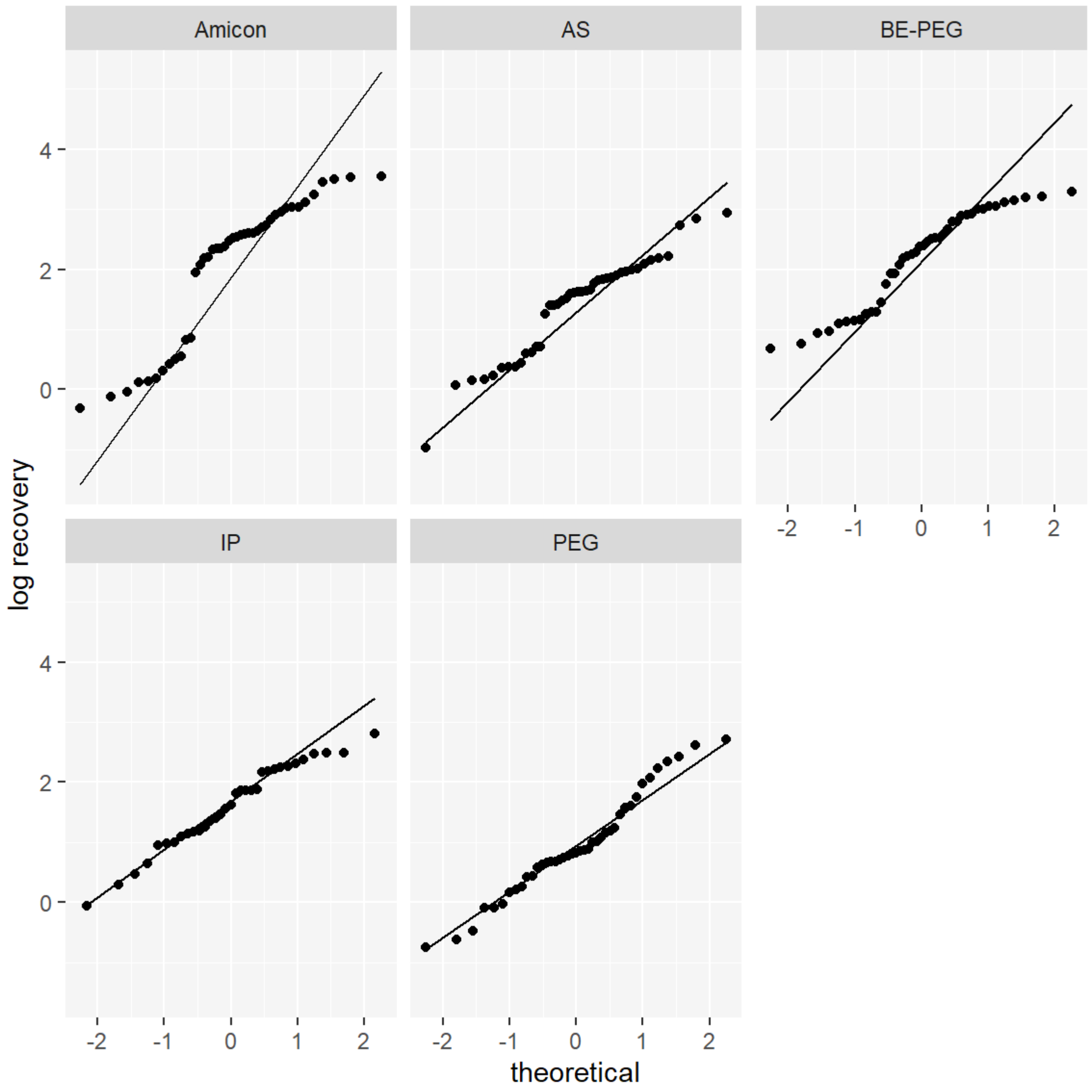

Figure S4: Quantile-quantile plot of log_{10} transformed virus recovery grouped by concentration method.

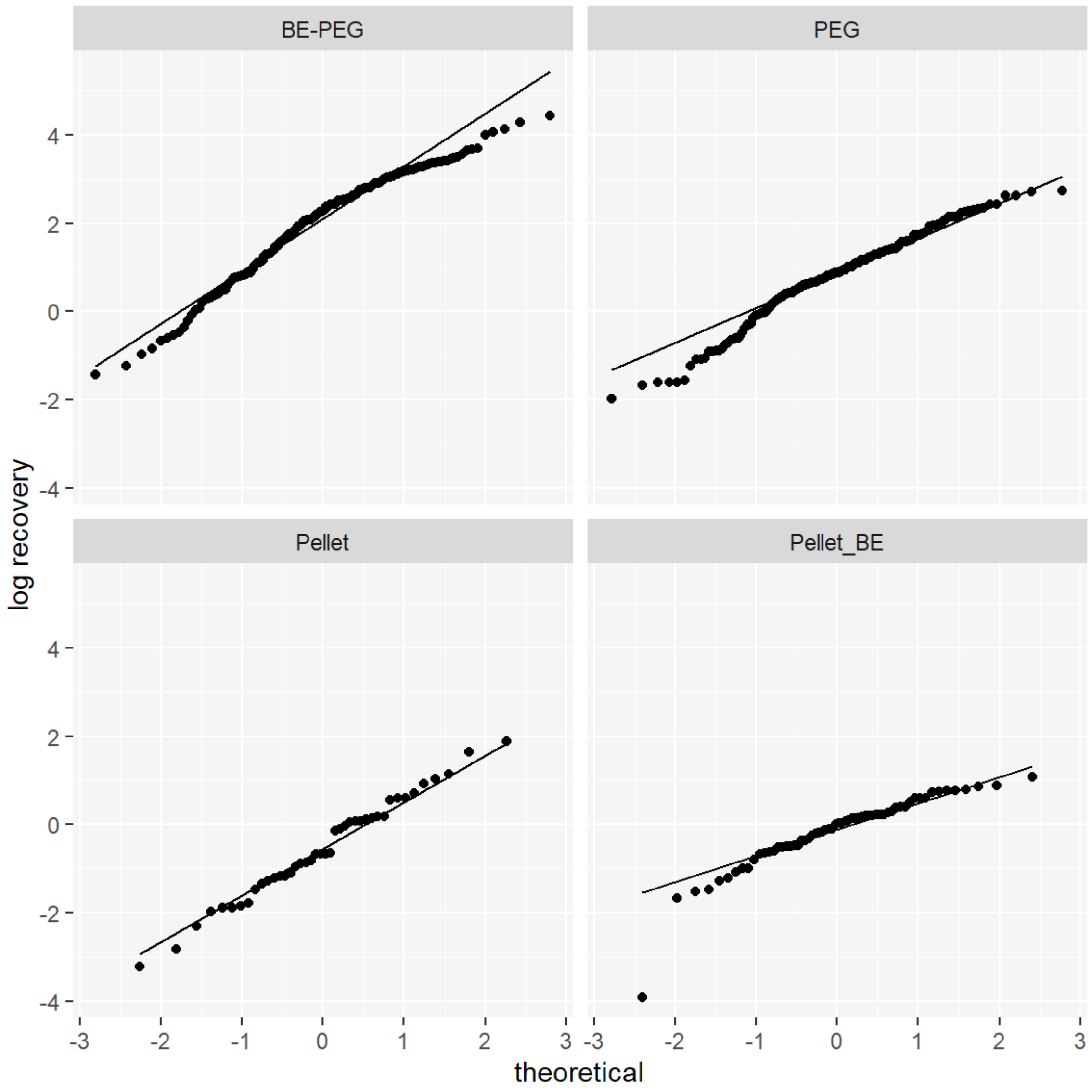

Figure S5: Quantile-quantile plot of log_{10} transformed virus recovery grouped by pellet and concentrated sample.

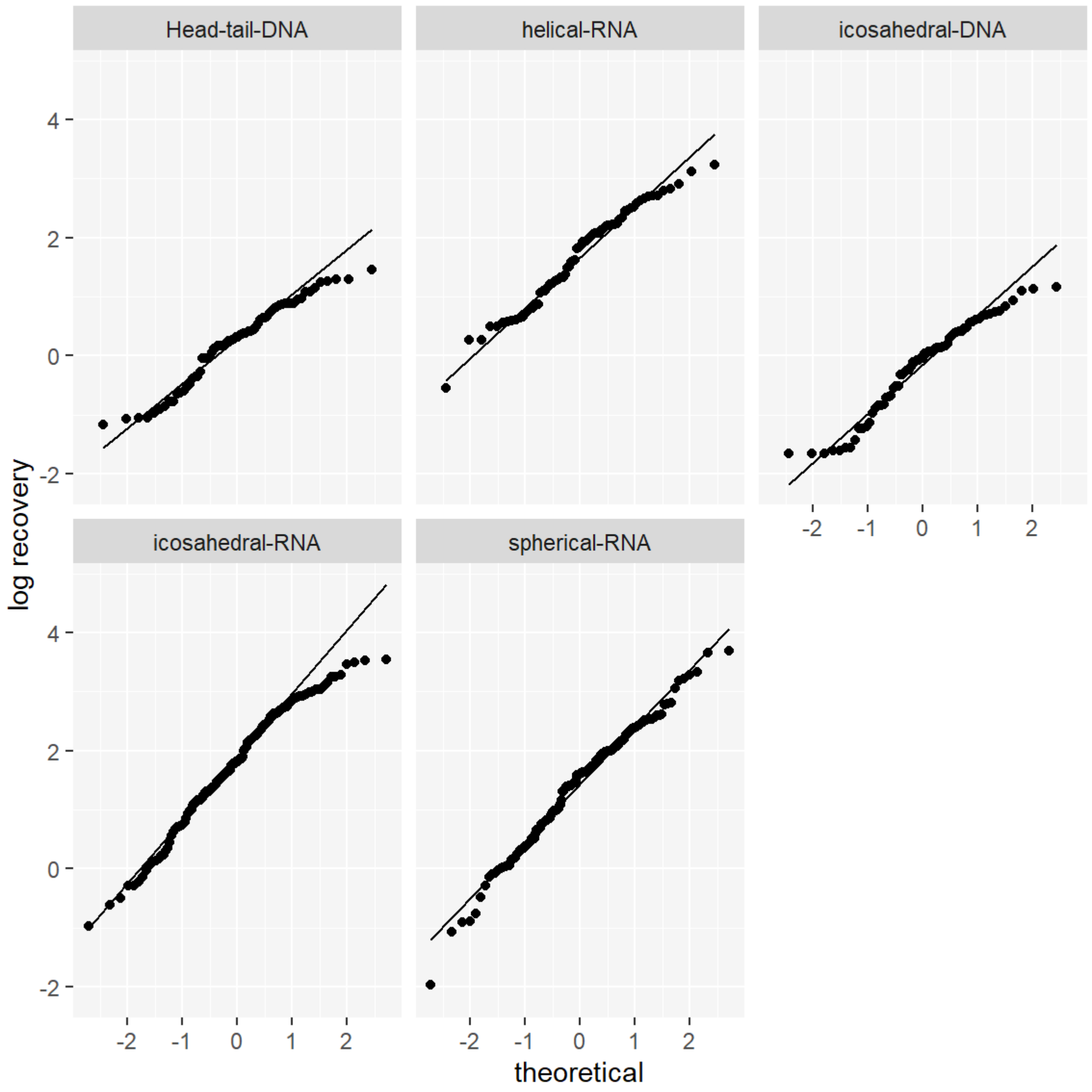

Figure S6: Quantile-quantile plot of log_{10} transformed virus recovery grouped by virus shape and genome type.

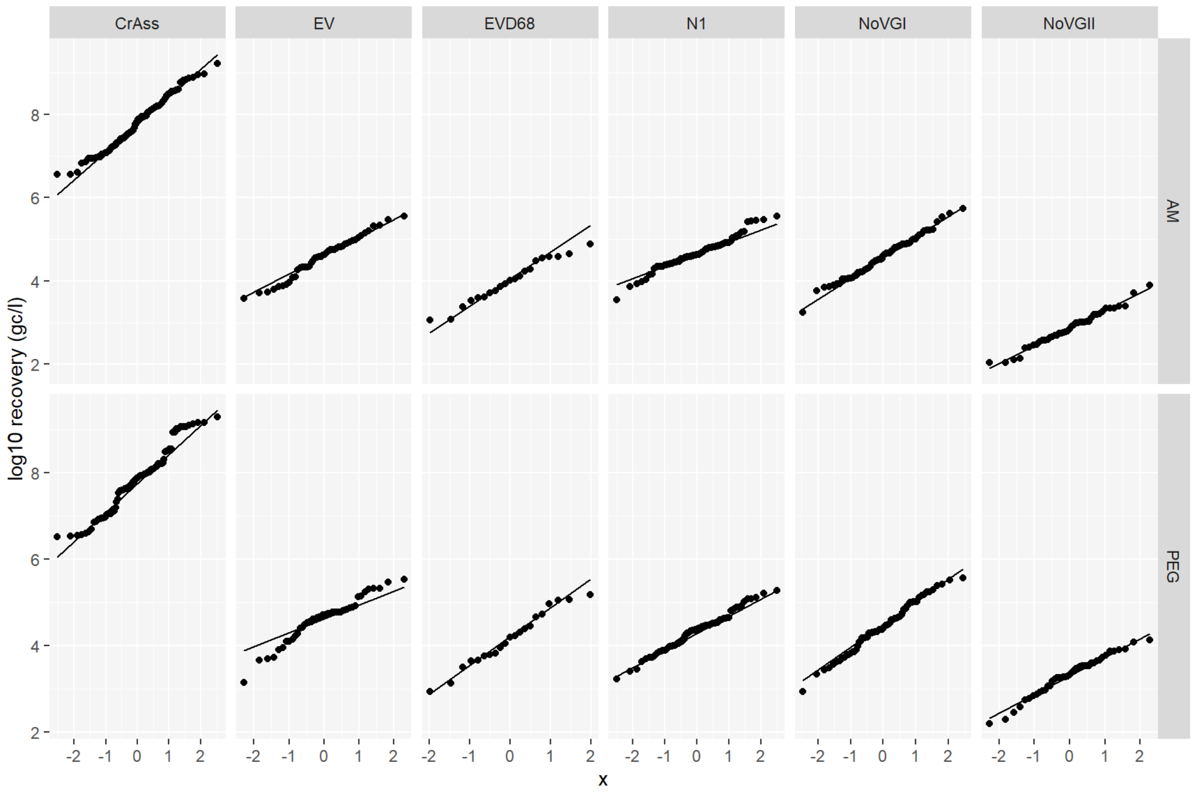

Figure S7. Quantile-quantile normality of the log_{10} transformed recovery of each virus with each concentration method.

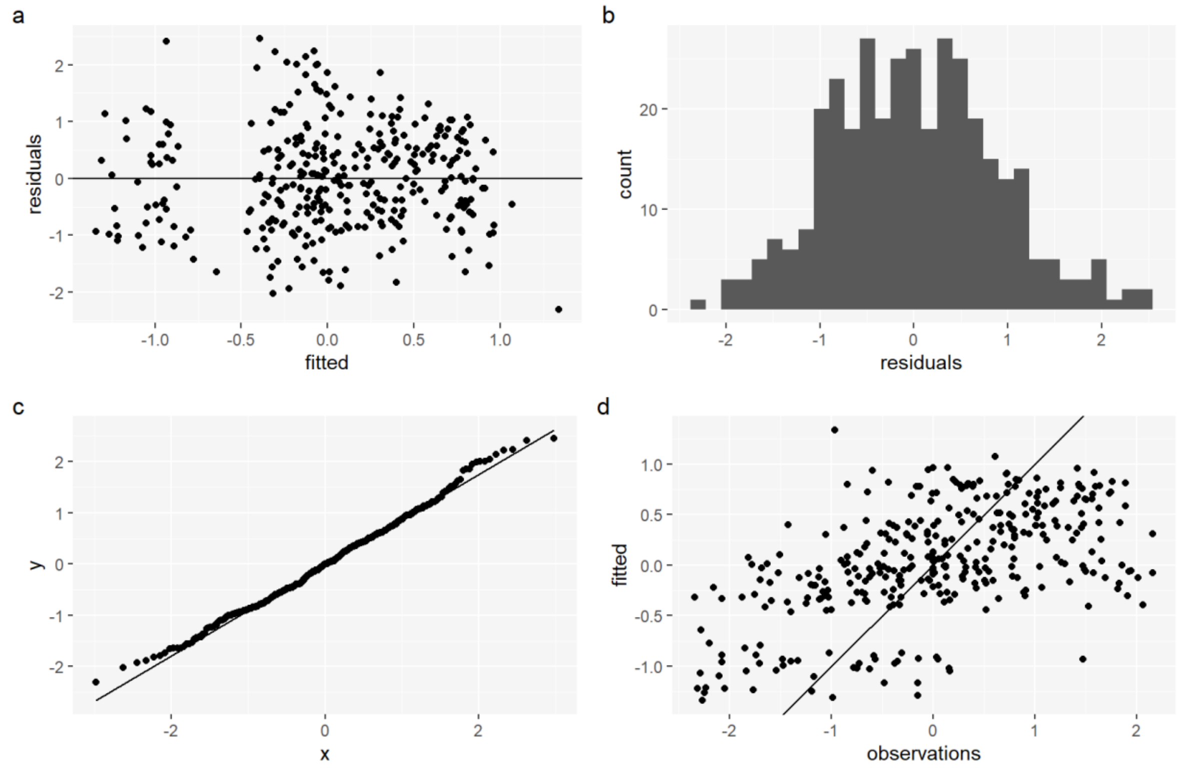

Figure S8. Residual plot of the linear mixed effects model. Residual plots of (a) homoscedasticity, (b) histogram normality, (c) quantile-quantile normality with x as a theoretical normal distribution, and (d) true observations against fitted values.

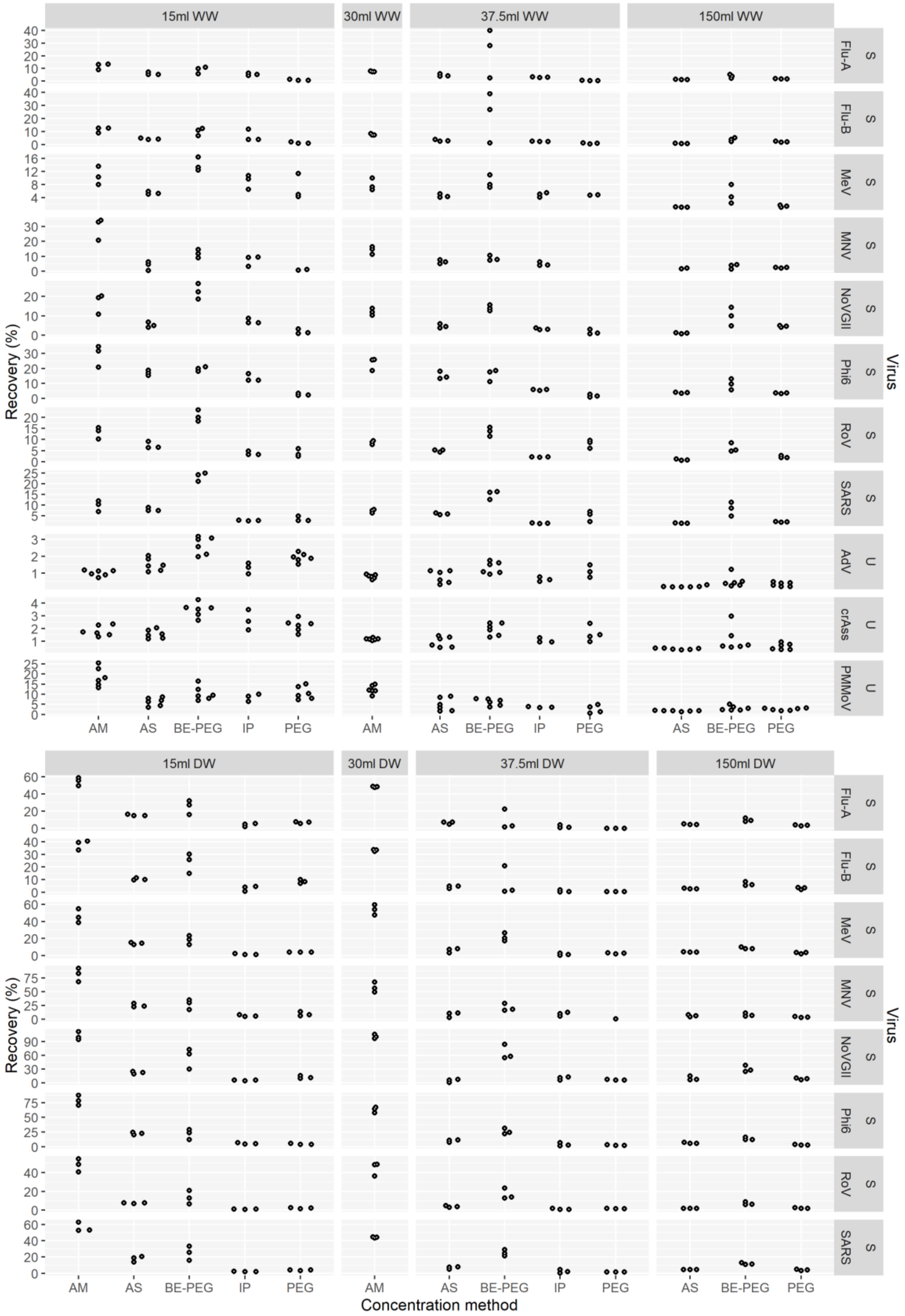

Figure S9. Recovery percentage for all 11 spiked (S) and unspiked (U) viruses grouped by wastewater (WW), deionised water (DW), sample volume (ml), and concentration method. Points have been offset on the horizontal axis to avoid over plotting.

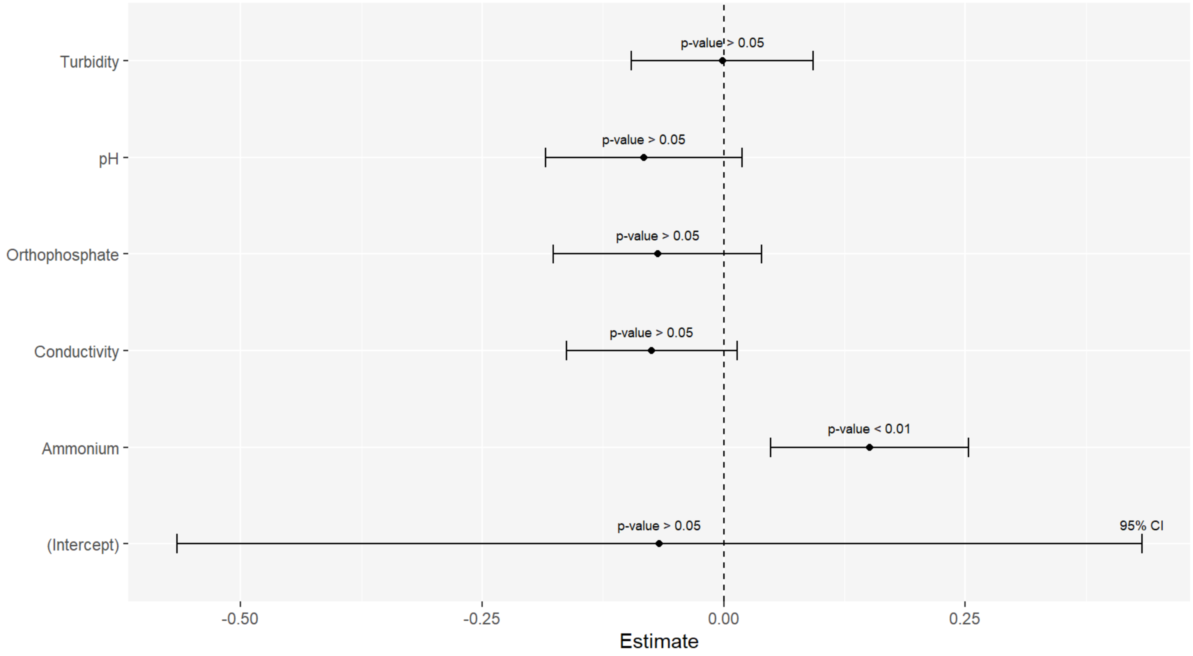

Figure S10: Linear mixed model assessing the effect of chemistry on the proportion of Amicon BE-PEG gene copies per litre recovery (Amicon recovery divided by BE-PEG recovery). The assessed proportion was power transformed using the Box-Cox method, selecting the lambda that maximised the log_{10}-Likelihood (lambda = -0.02). The target virus was included as random effects to account for the variability between viruses, borrowing strength to assess the effect of chemistry. Estimates less than zero suggest the Amicon method performs worse than the BE-PEG method when predictor variables increase, or on average with regards to the intercept. Estimates are labelled with their p-values and the line ranges around the estimate indicate the 95% confidence intervals (CI).

References

2019-Novel Coronavirus (2019-nCoV) Real-time rRT-PCR Panel Primers and Probes, 2020. . Centers for Disease Control and Prevention, Atlanta, Georgia.

Farkas, K., Malham, S.K., Peters, D.E., de Rougemont, A., McDonald, J.E., de Rougemont, A., Malham, S.K., Jones, D.L., 2017. Evaluation of two triplex one-step qRT-PCR assays for the quantification of human enteric viruses in environmental samples. Food Environ. Virol. 9, 343–349. https://doi.org/10.1007/s12560-017-9293-5

Gendron, L., Verreault, D., Veillette, M., Moineau, S., Duchaine, C., 2010. Evaluation of filters for the sampling and quantification of RNA phage aerosols. Aerosol Sci. Technol. 44, 893–901. https://doi.org/10.1080/02786826.2010.501351

Haramoto, E., Kitajima, M., Kishida, N., Konno, Y., Katayama, H., Asami, M., Akiba, M., 2013. Occurrence of pepper mild mottle virus in drinking water sources in Japan. Appl. Environ. Microbiol. 79, 7413–7418. https://doi.org/10.1128/AEM.02354-13

Hummel, K.B., Lowe, L., Bellini, W.J., Rota, P.A., 2006. Development of quantitative gene-specific real-time RT-PCR assays for the detection of measles virus in clinical specimens. J. Virol. Methods 132, 166–173. https://doi.org/https://doi.org/10.1016/j.jviromet.2005.10.006

ISO/TS 15216-2:2019-Microbiology of food and animal feed — Horizontal method for determination of hepatitis A virus and norovirus in food using real-time RT-PCR — Part 2: Method for qualitative detection, 2019. . International Organization for Standardization, Geneva Swizerland.

Kitajima, M., Oka, T., Takagi, H., Tohya, Y., Katayama, H., Takeda, N., Katayama, K., 2010. Development and application of a broadly reactive real-time reverse transcription-PCR assay for detection of murine noroviruses. J. Virol. Methods 169, 269–273. https://doi.org/10.1016/j.jviromet.2010.07.018

Poelman, R., Schölvinck, E.H., Borger, R., Niesters, H.G.M., van Leer-Buter, C., 2015. The emergence of enterovirus D68 in a Dutch University Medical Center and the necessity for routinely screening for respiratory viruses. J. Clin. Virol. 62, 1–5. https://doi.org/10.1016/j.jcv.2014.11.011

Stachler, E., Kelty, C., Sivaganesan, M., Li, X., Bibby, K., Shanks, O.C., 2017. Quantitative crAssphage PCR assays for human fecal pollution measurement. Environ. Sci. Technol. 51, 9146–9154. https://doi.org/10.1021/acs.est.7b02703

van Maarseveen, N.M., Wessels, E., de Brouwer, C.S., Vossen, A.C.T.M., Claas, E.C.J., 2010. Diagnosis of viral gastroenteritis by simultaneous detection of Adenovirus group F, Astrovirus, Rotavirus group A, Norovirus genogroups I and II, and Sapovirus in two internally controlled multiplex real-time PCR assays. J. Clin. Virol. 49, 205–210. https://doi.org/10.1016/j.jcv.2010.07.019
